# Circuit Specific Functions of Cannabinoid CB1 Receptor in the Balance of Investigatory Drive and Exploration

**DOI:** 10.1371/journal.pone.0026617

**Published:** 2011-11-01

**Authors:** Martin Häring, Nadine Kaiser, Krisztina Monory, Beat Lutz

**Affiliations:** Institute of Physiological Chemistry, University Medical Center of the Johannes Gutenberg University Mainz, Mainz, Germany; National Institutes of Health/NICHD, United States of America

## Abstract

Well balanced novelty seeking and exploration are fundamental behaviours for survival and are found to be dysfunctional in several psychiatric disorders. Recent studies suggest that the endocannabinoid (eCB) system is an important control system for investigatory drive. Pharmacological treatment of rodents with cannabinergic drugs results in altered social and object investigation. Interestingly, contradictory results have been obtained, depending on the treatment, drug concentration and experimental conditions. The cannabinoid type 1 (CB1) receptor, a central component of the eCB system, is predominantly found at the synapses of two opposing neuronal populations, i.e. on inhibitory GABAergic and excitatory glutamatergic neurons. In the present study, using different transgenic mouse lines, we aimed at investigating the impact of CB1 receptor inactivation in glutamatergic or GABAergic neurons on investigatory behaviour. We evaluated animate (interaction partner) and inanimate (object) exploratory behaviour in three different paradigms. We show that exploration was increased when CB1 receptor was deleted from cortical and striatal GABAergic neurons. No effect was observed when CB1 receptor was deleted specifically from dopamine receptor D1-expressing striatal GABAergic medium spiny neurons. In contrast, deletion of CB1 receptor from cortical glutamatergic neurons resulted in a decreased exploration. Thus, our results indicate that exploratory behaviour is accurately balanced in both, the social and non-social context, by the eCB system via CB1 receptor activation on cortical glutamatergic and GABAergic neurons. In addition, the results could explain the contradictory findings of previous pharmacological studies and could further suggest a possibility to readjust an imbalance in exploratory behaviour observed in psychiatric disorders.

## Introduction

Adequate novelty seeking and exploration are fundamental behaviours for survival. Dysfunctional exploratory profiles have been found in several distinct neuronal disorders, such as attention deficit disorder and schizophrenia-like diseases, expressed by modulated social behaviour and novelty seeking [1]–[5]. Thus, identifying control mechanisms of exploratory behaviour might allow new treatment strategies. Two recent studies indicated that the endocannabinoid (eCB) system might be important for a balanced response to novel situations [6], [7], but these studies elucidated only some aspects on the function of the eCB system in exploratory behaviour. Lafenêtre et al. [6] concentrated on object recognition with repeated exposures to a novel object and food pellet, thus, strongly reducing the novelty factor every day. Jacob et al. [7] performed multiple behavioural paradigms, including also social interaction studies. However, the latter study was only performed with animals lacking the cannabinoid type 1 (CB1) receptor completely or specifically in cortical glutamatergic neurons. To this end, the present study aimed at further detailing CB1 receptor functions in investigatory drive and exploration behaviour.

One important factor in exploratory behaviour is how a respective situation is evaluated. Brain regions involved in these evaluation processes, such as amygdala, hippocampus, and prefrontal cortex, show high levels of CB1 receptor mRNA and protein [8], [9]. These cortical areas possess two major neuronal subpopulations expressing the CB1 receptor; GABAergic interneurons (with high CB1 receptor levels) and glutamatergic neurons (with low CB1 receptor levels) [8], . The two neuronal populations represent the two major opposing players regarding the excitation state of the brain, namely GABAergic interneurons being inhibitory and glutamatergic neurons being excitatory. The endogenous ligands of CB1 receptor, the so-called endocannabinoids, are synthesized at the postsynapse and travel retrogradely to the CB1 receptor located at the presynapse [13]. Activated CB1 receptor then suppresses neurotransmitter release. Therefore, a functional eCB system may have a protective role to prevent an imbalance of neuronal activity and inadequate behavioural responses. In accordance with this notion, it was shown that the inactivation of the CB1 receptor gene from glutamatergic neurons leads to an increased vulnerability to kainic acid-induced seizures [10]. Furthermore, other behavioural studies indicated a bidirectional role of the eCB system in anxiety response based on CB1 receptor located on these two neuronal subpopulations [6], [7], [14].

Anxiety plays a critical role in exploratory and investigatory behaviour, and several pharmacological studies have shown the importance of the eCB system in social behaviour [15]–[19]. The results depended strongly on the treatment and experimental conditions, and they seemed to be contradictory at first sight. The acute and chronic administration of CB1 receptor agonists (Δ^9^-THC or WIN55,212-2), especially during adolescence, led to a decreased social interaction in rats. Opposing to this, treatment of adolescent rats with URB597, an inhibitor of anandamide degradation, or VDM11, a putative anandamide reuptake inhibitor, resulted in increased social play behaviour [17]–[19]. These latter findings are supported by studies with transgenic mice lacking the CB1 receptor ubiquitously or specifically in cortical glutamatergic neurons, where a decrease in object exploration and social interaction was shown, depending on the behavioural context [7], [20]. Altogether, these data suggest that strong systemic activation of the eCB system has anti-social effects, whereas on-demand enhancement of anandamide signalling and subsequent activation of CB1 receptor has a pro-social effect.

By using several conditional CB1 receptor knock-out mice, we aimed at investigating whether CB1 receptor on different neuronal cell types might explain the contradictory findings in social interaction and object exploration mentioned above. In order to address this question, we applied different behavioural paradigms to analyze inanimate (object) exploration and animate (interaction partner) exploration. Evaluating the results, we could detect a decreased exploratory drive in mice lacking CB1 receptor in cortical glutamatergic neurons. Mice lacking CB1 receptor in GABAergic neurons, including the striatum, displayed opposite results, namely, an increased exploratory drive. No changes in exploration were observed for mice lacking CB1 receptor specifically in striatal dopamine receptor D1-positive GABAergic medium spiny neurons. Thus, we hypothesize that cortical GABAergic interneurons are important for the increased exploratory drive. Altogether, our results suggest that exploratory behaviour (animate and inanimate) is balanced by the eCB system via CB1 receptor activation on the two opposing neuronal subpopulations.

## Materials and Methods

### Animals

This study was performed on adult (5–7 months old) male mutant mice and their respective wild-type littermates. Animals were housed in a temperature- and humidity-controlled room (22°C±1; 50%±1) with a 12 h light-dark cycle (lights on at 1 am) and had access to food and water *ad libitum*. The experimental protocols were carried out in accordance with the European Communities Council Directive of 24 November 1986 (86/609/EEC) and approved by the Ethical Committee on animal care and use of Rhineland-Palatinate, Germany. Generation, breeding and genotyping of the mutant lines were performed according to previous publications: CB1*^loxP/loxP;Nex-cre^* mice (referred to as Glu-CB1^−/−^ mice; [10]), CB1*^loxP/loxP;Dlx5/6-cre^* mice (referred to as GABA-CB1^−/−^ mice; [10], [21]), and CB1*^loxP/loxP;D1-cre^* mice (referred to as D1-CB1^−/−^ mice; [11]). While Glu-CB1^−/−^ mice lack the CB1 receptor in cortical glutamatergic neurons, GABA-CB1^−/−^ mice lack the CB1 receptor specifically in GABAergic neurons [10]. In D1-CB1^−/−^ mice, the CB1 receptor inactivation can primarily be found in GABAergic striatal medium spiny neurons, but also in a minor fraction of glutamatergic neurons in layer VI of the neocortex [11]. Wild-type littermates do not possess the respective Cre recombinase transgenic allele, and contain the CB1 floxed allele in a homozygous state. These mice were referred to as Glu-CB1^+/+^, D1-CB1^+/+^ and GABA-CB1^+/+^. All mutant lines were bred for >10 generations on the background of C57BL/6N mice from Charles River, Germany. For detailed information on the anatomical differences in CB1 receptor expression, see Monory et al. [11].

### Experimental design

Animals were group-housed (3–5 animals per cage type 2 (26.5×20.5×14.0 cm), EBECO Germany) until one week before behavioural testing. Animals were then separated and single- housed to avoid behavioural differences between dominant and subordinate animals. The same animals were used in each paradigm. Between each experimental paradigm, animals were allowed to rest for one week. All experiments were performed one hour after turning off the lights (2 pm), in the active phase of the animals, with only a minimal red light source in the room (0 lux).

### Open Field and Novel Object Recognition Task

The novel object recognition task combines a general exploration test with a visual recognition memory paradigm. Therefore, it is used to evaluate object exploration and object recognition. The test was performed in a white plastic open field chamber (H40 cm×W40 cm×L40 cm). The protocol used was modified from Ennaceur and Delacour , Tang et al., and Tordera et al. [22]–[24].

For habituation, the animals were placed into the empty open field and allowed to explore the box for 10 min once a day for two days. The first habituation session was analyzed according to a standard open field paradigm, hence, total distance moved and time spent in the center (defined as 20 cm×20 cm) was evaluated using SMART software (PanLab, Spain). On day 3, two identical objects (O1 left, and O1 right; two metal cubes with H4 cm×W3 cm×L5 cm) were placed symmetrically 6–7 cm from the walls and separated 16–18 cm from each other. The mouse was placed into the box at an equal distance from both objects and video-recorded for 10 min. After this first exposure to the object, the mouse was returned to its home cage. 2 h and 24 h later, the mouse was placed again into the open field and exposed to the familiar object (O1) and to a novel object (O2 for the 2 h time point, and O3 for the 24 h time point, respectively) each time for 10 min (retention tests). The novel object O2 was a plastic billiard ball (5.72 cm in diameter) fixed on a metal plate (0.2 cm) and O3 was a round glass flask (H6 cm×W3 cm), filled with sand and closed with a black rubber plug. The familiar object was always positioned on the left side, while the new object was on the right side. Box and objects were cleaned with 70% ethanol after each trial to avoid olfactory cues. Experiment was video-recorded and the total time that the animal spent exploring each of the two objects in training and retention phase was evaluated by an experimenter blind to the genotype. Object exploration was defined as the orientation of the nose directly to the object at a distance <2 cm and/or touching the object with the nose and whiskers. Time spent climbing and sitting on the object were not regarded as exploration, and was therefore excluded from measurement [22], as these activities do not present a form of exploration. The discrimination index (DI) was calculated as the difference between the time spent exploring the new (N) and familiar (F) object, divided by the total time exploring the objects [(N−F)/(N+F)]. A positive DI is considered to reflect increased memory retention for the familiar object [24].

### Sociability Test

A modified sociability test was performed, based on a published protocol [25]. In short, the test chamber (H41 cm×W42 cm×L70 cm) was divided into three compartments (H40 cm×W40 cm×L22 cm), all accessible by openings (H7.5 cm×W10 cm) in the dividing walls. Chambers and cages were cleaned with 70% ethanol between each trial to avoid olfactory cues. Experiment was video-recorded, and the total time that the test animals spent in each of the compartments during sociability and social novelty phase was measured by SMART software (PanLab, Spain). Male C57BL/6N animals (10–12 weeks old) were used as interaction partners for the sociability and social novelty phase.

#### Habituation Phase

The test animal was placed into the middle compartment for 5 min with entries to the side compartments blocked.

#### Sociability Phase

After the habituation phase, blockades of the entries were removed, allowing free access to the side compartments for 10 min. By doing this, the animal tested was exposed to a novel C57BL/6N interaction partner and a novel object (round cage described below), positioned in the two side compartments. The position of the interaction partner (left vs. right compartment) was alternated between trials to avoid any bias. The interaction partner itself was enclosed in a round cage (10 cm in diameter; 30 cm high [upper 20 cm Plexiglass, lower 10 cm covered by metal bars 1 cm apart to allow interaction but prevents fighting]). To minimize stress levels of the animals used as interaction partners, they were habituated to the cages four times for 10 min distributed over two days prior to the actual test days. To counterbalance individual differences of these interaction partners they were equally used for wild-type and mutant test mice. The novel object control (empty cage, no animal) was always positioned in the opposite compartment to the cage with the interaction partner. The discrimination index (DI) was calculated as the difference between the time spent exploring the novel object (nO) and the novel animal (nA), divided by the total time exploring both [(nO−nA)/(nO+nA)]. A positive DI is considered to reflect increased preference for the social interaction partner.

#### Social Novelty Phase

2 h after the sociability phase, an additional, unknown interaction partner (novel) was introduced. The interaction partner from the sociability phase (familiar) was again placed into the same cage and same compartment as before. The novel animal was placed into the former empty cage and positioned at the respective side compartment. Openings were unblocked. The test animal was placed into the middle compartment, and the test animal was allowed to freely explore for 10 min. The DI was calculated as the difference between the time spent exploring the new (N) and the familiar (F) animal, divided by the total time exploring both [(N−F)/(N+F)]. A positive DI is considered to reflect increased memory retention for the familiar animal.

### Resident-Intruder Test

The resident-intruder test was performed by placing a novel, group-housed intruder into the home cage of the test animal for 10 min. This paradigm allows evaluating social exploration and aggressive behaviour [26]. To decrease interaction induced by the intruder, younger animals (males, 11–13 weeks) were used as intruders. Experiment was video-recorded, and the total interaction time of the animals spent exploring was measured by an experimenter blind to the genotype. Interaction was defined by any type of physical interaction induced by the resident clearly directed towards the partner. Duration, percentage of time and number of fights were evaluated separately. Fighting was defined by physical struggling between the interaction partners initiated by an attack of the resident towards the intruder.

### Statistical analysis

Data are presented as mean ± standard error of the mean (SEM) of individual data points. Results were considered to be significant at p<0.05. All behavioural endpoints of the novel object recognition task were initially analyzed using two-way ANOVA, using genotype and object as variables and Bonferroni post-tests to correct for multiple comparisons. In some cases, to analyze the locomotion effects in the open field, the sociability in the sociability test and the aggression in the resident-intruder paradigm for each genotype, data were analysed using an unpaired Student's t-test or Kruskal-Wallis statistic. Additionally, in order to evaluate whether the DI of the genotypes deviated significantly from zero, we used the unpaired t-test with Welch's correction. Graphs and statistics were generated by GraphPad Prism 4.03 (GraphPad Software; http://www.graphpad.com).

## Results

### Open Field

The evaluation of the locomotor activity in the open field revealed that only the GABA-CB1^−/−^ mice showed an alteration (T_(18)_ = 3.213, p = 0.0048; Table 1). None of the other mutants showed any change in the distance moved compared with their respective wild-type littermates in the open field (Glu-CB1 line [T_(34)_ = 1.609, p = 0.1169]; D1-CB1 line [T_(21)_ = 0.5618, p = 0.5802]). In regard to the time spent in the center region of the open field, we could not detect an alteration in any of the mutants (Glu-CB1 line [T_(34)_ = 0.8168, p = 0.4197]; GABA-CB1 line [T_(18)_ = 1.418, p = 0.1733]; D1-CB1 line [T_(21)_ = 0.9048, p = 0.3758]; see Table 1).

**Table 1:** Locomotion, anxiety and memory.

	Glu-CB1		GABA-CB1		D1-CB1	
Paradigm	+/+	−/−	+/+	−/−	+/+	−/−
	**Distance Moved (cm)**					
**Open Field**	2824±209	2324±182	2621±306	3801±202**	4456±91	4368±131
**Sociability**						
Habituation	2012±113	1569±162*	1597±59	1730±64	1669±81	1850±118
Sociability	5171±205	4891±312	4945±127	5083±175	5055±162	5079±157
Social Novelty	3973±211	3125±227*	4023±141	4623±175*	3863±292	3942±160
	**Time in Center (sec)**					
**Open Field**	63.3 ± 12	80.9±20	156.0±48	82.0±21	65.4±11	82.4±15
	**Discrimination Index (DI)**					
**NORT**						
Training	0.01±0.01	−0.08±0.08	−0.03±0.03	−0.03±0.02	−0.08±0.05	0.03±0.02
Retention 2 h	0.16±0.03#	0.06±0.05	0.00±0.06	−0.03±0.03	0.15±0.06#	0.08±0.03#
Retention 24 h	0.25±0.06#	0.06±0.13	0.18±0.06#	0.15±0.05#	0.27±0.08#	0.18±0.05#
**Sociability**						
Sociability	0.29±0.03#	0.12±0.07*	0.27±0.03#	0.35±0.04#	0.20±0.04#	0.30±0.04#
Social Novelty	0.05±0.03	0.08±0.09	−0.01±0.05	0.09±0.03#	0.03±0.06	0.03±0.06

Evaluation of locomotion (distance moved), anxiety (time in center) and memory (discrimination index) for all mutant lines; ^+/+^ (wild-type), ^−/−^ (mutant); t-test analysis:

*p<0.05;

**p<0.01 (significance between genotype);

# p<0.05 (significant from 0; positive recognition of novel object).

### Novel Object Recognition Task

**Figure 1:**
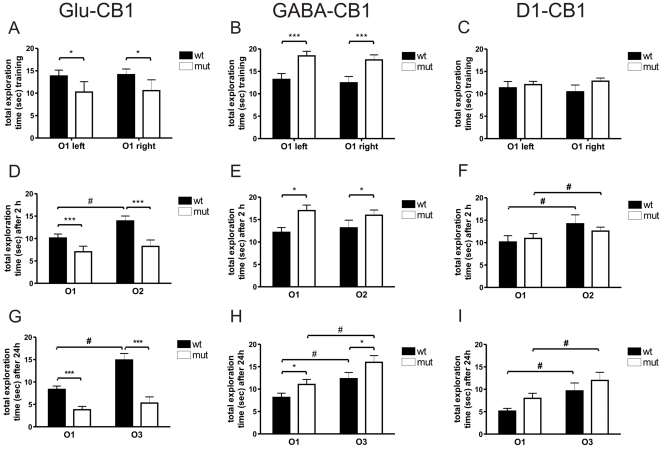
Inanimate exploration in the novel object recognition task. (A–C) Total time of exploration of two identical objects (O1, both on left and right side) during the training session for three conditional CB1 receptor mutant lines (Glu-CB1 [n = 23+13], GABA-CB1 [n = 18+23], D1-CB1 [12+12]) and their wild-type control littermates. (D–F) Total time of exploration of familiar object (O1) and novel object (O2 or O3) during the retention session after 2 h or 24 h (G–I). Glu-CB1^−/−^ mice displayed a reduced exploration, while GABA-CB1^−/−^ mice showed an increased exploration both in the training and retention session when compared to their wild-type littermate controls. No significant genotype differences were observed in the D1-CB1 mutant line. 2-way ANOVA (genotype differences) *p<0.05, ***p<0.001; t-test (discrimination index DI) ^#^p<0.05.

The analysis of the novel object recognition task (referred to as NORT in Table 1) revealed a decrease in general object exploration in Glu-CB1^−/−^ mice as compared to wild-type littermate controls (Fig. 1A,D,G). We detected a significant decrease in time spent with the objects O1 in the training session (F_(1,62)_ = 4.183, p = 0.0451; Fig. 1A), but also in the 2 h (F_(1,66)_ = 13.68, p = 0.0004; Fig. 1D) and 24 h retention sessions (F_(1,66)_ = 32.87, p<0.0001; Fig. 1G) for the novel object O2. In contrast, GABA-CB1^−/−^ mice displayed a general increase in exploration in all the sessions as compared to controls (training [F_(1,74)_ = 17.88, p<0.0001], 2 h retention [F_(1,74)_ = 8.411, p = 0.0049)], 24 h retention [F_(1,74)_ = 6.172, p = 0.0152]; Fig. 1B,E,H). In the D1-CB1 mutant line, no genotype differences were observed in the general object exploration (training [F_(1,44)_ = 1.760, p = 0.1915], 2 h retention [F_(1,44)_ = 0.08051, p = 0.7721], 24 h retention [F_(1,44)_ = 3.317, p = 0.0754]; Fig. 1C,F,I).

Evaluation of the discrimination index (DI) revealed that all groups, independent of the line, showed no differences within the training session regarding the exploration of the left and the right object O1, respectively. (Glu-CB1^+/+^ [T_(20)_ = 0.8230, p = 0.4202]; Glu-CB1^−/−^ [T_(11)_ = 0.9582, p = 0.3585]; GABA-CB1^+/+^ [T_(15)_ = 1.118, p = 0.2812]; GABA-CB1^−/−^ [T_(22)_ = 1.959, p = 0.0630]; D1-CB1^+/+^ [T_(11)_ = 1.447, p = 0.1758]; D1-CB1^−/−^ [T_(11)_ = 1.679, p = 0.1213]; Table 1). Furthermore, no discrimination differences compared to their respective wild-type controls were found for all mutants within the training session (Glu-CB1 line [T_(31)_ = 1.407, p = 0.1693]; GABA-CB1 line [T_(37)_ = 0.06488, p = 0.9486]; D1-CB1 line [T_(22)_ = 1.951, p = 0.0639]; Table 1).

In the 2 h retention phase, several groups lacked a significant discrimination between the familiar and the novel object. Only Glu-CB1^+/+^, D1-CB1^+/+^ and D1-CB1^−/−^ animals displayed a significant preference towards the novel stimulus (Glu-CB1^+/+^ [T_(21)_ = 4.806, p<0.0001]; Glu-CB1^−/−^ [T_(12)_ = 1.220, p = 0.2458]; GABA-CB1^+/+^ [T_(15)_ = 0.07097, p = 0.9444]; GABA-CB1^−/−^ [T_(22)_ = 1.366, p = 0.1858]; D1-CB1^+/+^ [T_(10)_ = 2.502, p = 0.0313]; D1-CB1^−/−^ [T_(10)_ = 2.238, p = 0.0492]; Table 1). Comparison between the mutants and their respective wild-type littermates displayed no significant differences in all lines (Glu-CB1 line [T_(33)_ = 1.775, p = 0.0850]; GABA-CB1 line [T_(37)_ = 0.6235, p = 0.5368]; D1-CB1 line [T_(20)_ = 0.9965, p = 0.3309]; Table 1).

In the 24 h retention phase, independently of the genotype, all groups showed a significant preference towards the novel object, with the only exception of the Glu-CB1^−/−^ animals (Glu-CB1^+/+^ [T_(21)_ = 4.472, p = 0.0002]; Glu-CB1^−/−^ [T_(12)_ = 0.4328, p = 0.6729]; GABA-CB1^+/+^ [T_(15)_ = 2.818, p = 0.0129]; GABA-CB1^−/−^ [T_(22)_ = 3.072, p = 0.0056]; D1-CB1^+/+^ [T_(11)_ = 3.601, p = 0.0042]; D1-CB1^−/−^ [T_(11)_ = 3.540, p = 0.0046]; Table 1). Comparison between the mutants and their respective wild-type littermates diplayed no genotype difference (Glu-CB1 line [T_(33)_ = 1.522, p = 0.1374]; GABA-CB1 line [T_(37)_ = 0.1255, p = 0.9008 ]; D1-CB1 line [T_(22)_ = 1.049, p = 0.3055]; Table 1).

The evaluation of object specific exploration (O1 left or O1-3 right) over the three sessions (training, 2 h retention and 24 h retention), revealed a significant difference for the Glu-CB1^−/−^ as compared to their littermate controls. Thus, the Glu-CB1^−/−^ mutants showed a steadily decreasing investigatory behaviour for both, the left object (increasing familiarity) and the right object (always novel) (Glu-CB1^−/−^ interaction [object/time]: F_(2,48)_ = 0.1537, p = 0.8580; Bonferroni post-test: training p>0.05, 2 h p>0.05, 24 h p>0.05; Fig. 1A,D,G). This phenomenon was only seen in the Glu-CB1^+/+^ mice for the left object (increasing familiarity), while the time spent investigating the right object (always novel) remained constant (Glu-CB1^+/+^ interaction [object/time]: F_(2,84)_ = 4.851, p = 0.0101; Bonferroni post-test: training p>0.05, 2 h p>0.05, 24 h p<0.01; Fig. 1A,D,G). It was further possible to detect a significant difference between the genotypes in exploring the right object, but not the left object over the three sessions (left object interaction [genotype/time]: F_(2,48)_ = 0.2283, p = 0.7965; Bonferroni post-test: training p>0.05, 2 h p>0.05, 24 h p>0.05; right object interaction [genotype/time]: F_(2,66)_ = 3.522, p = 0.0352; Bonferroni post-test: training p>0.05, 2 h p<0.05, 24 h p<0.001; Fig. 1A,D,G).

### Sociability Test

**Figure 2:**
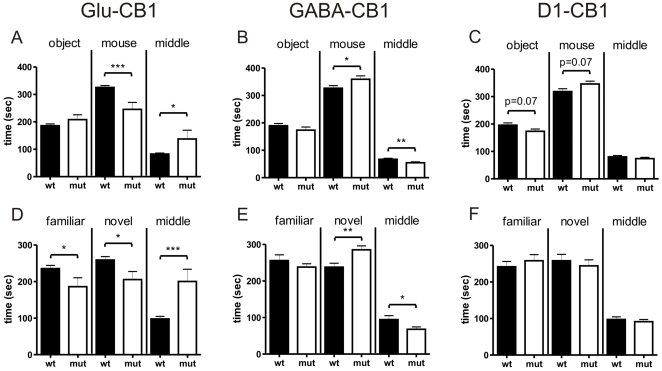
Animate vs. inanimate exploration in the sociability test. (A–C) Comparison of animate (mouse) and inanimate (object, “empty”) exploration for the three mutants lines (Glu-CB1 [n = 22+13], GABA-CB1 [n = 18+23], D1-CB1 [16+16]) and their wild-type littermate controls during the sociability phase. (D–F) Exploration of the familiar and the novel interaction partner for during the social novelty phase. Glu-CB1^−/−^ mice displayed no significant change in the exploration session, where there was a choice between the object and the interaction partner. In the social novelty phase, however, the interaction with a novel interaction partner was decreased when compared with their wild-type littermate controls. GABA-CB1^−/−^ mice showed an increased social interaction in both sessions. In the D1-CB1 mutant line, no genotype differences were observed neither in the sociability nor in the social novelty phase. n = 11–20 animals; t-test *p<0.05, **p<0.01.

During the sociability phase, the Glu-CB1^−/−^ animals showed a significant increase in time spent in the middle compartment (T_(33)_ = 2.247, p = 0.0314; Fig. 2A). Accordingly, these mutants displayed a significant decrease in time spent with the interaction partner but not with the object (mouse [T_(33)_ = 3.734, p = 0.0007]; object [T_(33)_ = 1.412, p = 0.1672]; Fig. 2A). A similar result was obtained, when the novel interaction partner was introduced during the social novelty test. While the Glu-CB1^−/−^ mice spent more time in the middle compartment, they spent less time with the familiar and novel partner as compared to the wild-type littermates (middle [T_(33)_ = 3.772, p = 0.006]; familiar [T_(33)_ = 2.263, p = 0.0303]; unknown [T_(33)_ = 2.596, p = 0.0140]; Fig. 2D). This phenotype was opposite to the findings with the GABA-CB1 line. In the sociability phase as well as in the social novelty phase, the GABA-CB1^−/−^ mice showed a significant increase in time spent with the novel interaction partner as compared to controls (sociability [T_(57)_ = 2.099, p = 0.0403]; social novelty [T_(35)_ = 3.063, p = 0.0042]; Fig. 2B,E). The time spent in the middle compartment was consequently decreased (sociability [T_(57)_ = 2.740, p = 0.0082]; social novelty [T_(35)_ = 2.168, p = 0.037]). Interestingly, the time spent in the compartment with the empty cage (i.e. the object only) during the sociability phase as well as the time spent with the familiar animal (social novelty test) were not different between mutants and controls (object [T_(57)_ = 1.114, p = 0.2699]; familiar [T_(35)_ = 1.017, p = 0.3162]; Fig. 2B,E). The analysis of the D1-CB1 line did not reveal any significant genotype differences in the 3 phases of the sociability test (Fig. 2C,F). Only a non-significant trend was observed in the sociability phase. Even though time spent in the middle area was not altered (T_(28)_ = 0.9190, p = 0.3659), it seemed that the mutants showed a slight preference to explore the interaction partner (T_(28)_ = 1.909, p = 0.0666) rather than the empty cage (T_(28)_ = 1.859, p = 0.0736), i.e. the object (Fig. 2C). In the social novelty phase, not even a trend was detectable (familiar [T_(30)_ = 0.7636 p = 0.4511]; novel [T_(30)_ = 0.5840 p = 0.5636]; middle [T_(30)_ = 0.6112 p = 0.5457]; Fig. 2F).

The evaluation of the DI showed only minimal differences between the genotypes. In the sociability phase, the Glu-CB1^−/−^ animals showed an impaired preference towards the interaction partner as compared to their controls (T_(33)_ = 2.537, p<0.0161; Table 1). In contrast, the GABA-CB1^−/−^ mice and the D1-CB1^−/−^ mice showed no significant changes in the preference towards the interaction partner (GABA-CB1^−/−^ [T_(57)_ = 1.507, p<0.1373], D1-CB1^−/−^ [T_(28)_ = 1.636, p<0.1130]; Table 1). In the social novelty phase, no DI differences were observed in any of the lines (Glu-CB1 line [T_(33)_ = 0.3977 p = 0.6934]; GABA-CB1 line [T_(34)_ = 1.794 p = 0.0817]; D1-CB1 line [T_(30)_ = 0.6126 p = 0.547]).

For all lines and genotypes, except for the Glu-CB1^−/−^ mice, we observed a strong preference towards the social interaction partner over the object in the sociability phase (Glu-CB1^+/+^ [T_(21)_ = 10.47, p<0.0001]; Glu-CB1^−/−^ [T_(12)_ = 1.559, p = 0.1450]; GABA-CB1^+/+^ [T_(27)_ = 8.309, p<0.0001]; GABA-CB1^−/−^ [T_(30)_ = 8.187, p<0.0001]; D1-CB1^+/+^ [T_(16)_ = 5.017, p = 0.0002]; D1-CB1^−/−^ [T_(13)_ = 7.458, p<0.0001]; Table 1). In the social novelty phase, none of the groups, except for the GABA-CB1^−/−^ mice, showed any preference towards the novel over the familiar interaction partner (Glu-CB1^+/+^ [T_(21)_ = 1.453, p<0.1610]; Glu-CB1^−/−^ [T_(12)_ = 0.8652, p = 0.4039]; GABA-CB1^+/+^ [T_(15)_ = 0.2402, p = 0.8134]; GABA-CB1^−/−^ [T_(19)_ = 2.674, p = 0.0150]; D1-CB1^+/+^ [T_(16)_ = 0.4262, p = 0.6756]; D1-CB1^−/−^ [T_(14)_ = 0.4437, p = 0.6841]; see Fig. 1).

The evaluation of the locomotor activity revealed no significant changes in the habituation phase of the sociability test, except for the Glu-CB1^−/−^ mice, which showed a decrease in locomotion (Glu-CB1 line [T_(33)_ = 2.312, p = 0.0271]; GABA-CB1 line [T_(60)_ = 1.506, p = 0.1374]; D1-CB1 line [T_(29)_ = 1.571, p = 0.1270]). In the sociability phase, no alteration in the distance moved was observed in any of the lines (Glu-CB1 line [T_(29)_ = 0.7833, p = 0.4398]; GABA-CB1 line [T_(62)_ = 0.6159, p = 0.5402]; D1-CB1 line [T_(30)_ = 0.1082, p = 0.9145]). However, a significant decrease and increase in the distance moved was detected in the social novelty phase for the Glu-CB1^−/−^ mice and the GABA-CB1^−/−^ mice, respectively (Glu-CB1 line [T_(33)_ = 2.575, p = 0.0146]; GABA-CB1 line [T_(38)_ = 2.591, p = 0.0135]). The D1-CB1^−/−^ mice again showed no change in the distance moved as compared to their respective wild-type littermates (T_(30)_ = 0.2386, p = 0.8130).

### Resident-Intruder Test

**Figure 3:**
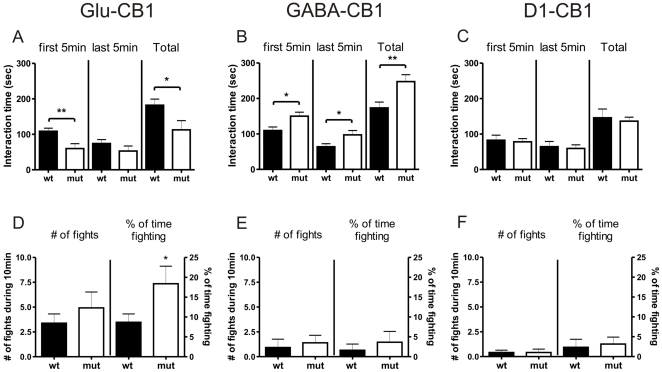
Animate exploration in the resident-intruder test. (A–C) Social interaction with an unknown, younger intruder for all three mutant lines (Glu-CB1 [n = 23+13], GABA-CB1 [n = 18+23], D1-CB1 [n = 16+16]). (D–E) Number of fights induced by the resident is shown for all three mutant lines. Glu-CB1^−/−^ mice showed a significantly reduced exploration during the first 5 min observation period and an increased aggression towards the intruder when compared to wild-type littermate controls. GABA-CB1^−/−^ mice displayed an increased interaction with the intruder, but no difference in aggressive behaviour. D1-CB1^−/−^ mice showed no behavioural changes as compared to their wild-type littermate controls. t-test *p<0.05, **p<0.01.

Glu-CB1^−/−^ mice displayed a significant decrease interacting with the intruder animals for the 10 min interaction phase as compared with wild-types (T_(35)_ = 2.297, p = 0.0277). Splitting the 10 min period into two 5 min bins revealed that the difference in interaction was mainly visible for the first 5 min bin (T_(35)_ = 3.106, p = 0.0038) (Fig. 3A). In addition, Glu-CB1^−/−^ mice displayed an altered aggressive behaviour. Even though the number of fights was not different between the genotypes, the time that Glu-CB1^−/−^ mice spent fighting the intruder was increased (T_(35)_ = 2.249, p = 0.0309) (Fig. 3D). As observed in the previous experiments, we detected an opposite phenotype in the GABA-CB1^−/−^ animals, which showed an increased interaction with the intruder animal (T_(29)_ = 2.522, p = 0.0174) (Fig. 3B). The overall fighting with the younger intruder did not change as compared to the wild-type littermates (T_(26)_ = 0.4227, p = 0.6760, T_(29)_ = 0.6286, p = 0.5345) (Fig. 3E). D1-CB1^−/−^ mice again displayed no phenotype differences, neither in interaction time spent with the intruder (T_(20)_ = 0.3481, p = 0.7314), nor in fighting behaviour (T_(22)_ = 0.0000, p = 1.0, T_(22)_ = 0.8261, p = 0.4176) (Fig. 3C,F).

Additional analysis revealed that Glu-CB1^+/+^ animals displayed a significant increase in aggression as compared to the other control groups, GABA-CB1^+/+^ and D1-CB1^+/+^. Thus, differences were detected in number of fights (Kruskal-Wallis statistic = 7.478, p = 0.0238; Dunn's Multiple Comparison Post-Test: Glu-CB1^+/+^ vs GABA-CB1^+/+^ p<0.05, Glu-CB1^+/+^ vs D1-CB1^+/+^ p>0.05, GABA-CB1^+/+^ vs D1-CB1^+/+^ p>0.05), as well as % of time fighting (Kruskal-Wallis statistic = 7.584, p = 0.0226; Dunn's Multiple Comparison Post-Test: Glu-CB1^+/+^ vs GABA-CB1^+/+^ p<0.05, Glu-CB1^+/+^ vs D1-CB1^+/+^ p>0.05, GABA-CB1^+/+^ vs D1-CB1^+/+^ p>0.05).

## Discussion

Using different conditional CB1 receptor mutant mice, we were able to show that the deletion of the CB1 receptor from forebrain GABAergic or cortical glutamatergic neurons resulted in an opposite behavioural outcome regarding animate and inanimate exploration. On the other hand, deletion of the CB1 receptor from dopamine receptor D1-expressing GABAergic striatal medium spiny neurons did not result in any significant changes. These findings suggest a regulatory function of the eCB system in cortical GABAergic and glutamatergic circuits to prevent neuronal and behavioural imbalance.

Mice lacking the CB1 receptor on glutamatergic neurons displayed a decreased exploratory behaviour, both in animate interaction (the interaction with a partner) and inanimate interaction (the interaction with an object). A similar decrease in object and social exploration was found in earlier studies, which were related with increased fear [6], [7]. In our study, the decrease in exploration was seen when the mouse was exposed to a social interaction partner and/or to an object, and seemed to be independent of novelty (Figs. 1, 2D). This phenotype was also visible in the resident-intruder test. However, the decreased social investigation was mainly based on a lower exploration during the first 5 min interval, a period important for information gathering (Fig. 3A).

The anxiogenic-like behaviour associated to these mutants can also explain the significantly higher aggression level found in the resident-intruder paradigm (Fig. 3D), a behaviour which was also observed in complete CB1 receptor knock-out animals [27]. The age and strength of the intruder compared to the resident is highly important [28]. In our case, the intruders were weaker and should not be regarded as a threat. We would therefore suggest that the deletion of the CB1 receptor from glutamatergic neurons might result in an inadequate aggressive response, suggesting an important role of CB1 receptor on this neuronal population in aggression. CB1 receptor in cortical GABAergic interneurons appears to mediate an opposite behaviour. While D1-CB1^−/−^ animals (CB1 receptor loss primarily in the striatum), did not reveal any significant difference as compared to wild-type littermates, we observed that GABA-CB1^−/−^ mice (lacking CB1 receptor additionally from cortical GABAergic interneurons), showed an increase in animate and inanimate exploration. Accordingly, increased investigatory behaviour toward novel food or object was previously observed in the GABA-CB1^−/−^ mice [6]. Interestingly, Glu-CB1^+/+^ control animals displayed an increased aggressive behaviour in the resident intruder paradigm as compared to the other wild-type controls, GABA-CB1^+/+^ and D1-CB1^+/+^. This elevated aggression might be explained by the fact that both the wild-type and mutant littermates are group-housed during growth. In case of the Glu-CB1 line, the modulated social behaviour of the Glu-CB1^−/−^ mutants might have an effect on their wild-type littermates.

Taken together, these results suggest an anxiolytic-like function of the CB1 receptor on glutamatergic neurons and an anxiogenic-like function of the CB1 receptor on GABAergic interneurons. However, a generalized conclusion on the involvement CB1 receptor on cortical glutamatergic neurons in anxiety is not yet possible to be drawn, as under our experimental conditions, the open field test was not congruent with this notion. Neither Glu-CB1^−/−^ nor GABA-CB1^−/−^ mutants spend a different period of time in the more aversive center zone as compared with their respective wild-type littermates (Table 1). In addition, studies with these animals on the elevated plus maze, an anxiety test, did not reveal any changes either [7], [14]. Also, levels of corticosterone under basal and stressful conditions were found to be similar between mutant and wild-type controls in both mutant lines [29]. Thus, it seems that a respective exploratory stimulus, such as an object or interaction partner, is required to induce a phenotype in these mice.

An alternative explanation for the observed differences can be alterations in spontaneous locomotor activity. In fact, we observed for both the Glu-CB1^−/−^ and GABA-CB1^−/−^ changes in the distance moved, namely a decrease and increase, respectively. It seems unlikely that the difference in locomotion was the driving force underlying the exploration phenotypes, as the mutants, in contrast to the variation in animate and social investigation, did not always display the locomotor alterations (Table 1). We argue that a respective context (e.g. handling threshold, exploratory stimulus) is required for a detectable locomotion phenotype in our mutant lines. A similar situation seems to be true for the general investigatory drive. Thus, the clear differences in exploring object or interaction partner is not mirrored by the findings in the open field test, where we were not able to detect any alteration in the time spent in the more aversive center region (Table 1). This notion is supported by other studies with these mutant lines, where a behavioural change is only detectable in the presence of a respective stimulus or pharmacological modification of the eCB system [6], [7].

A further explanation for the behavioural differences might be memory alterations in the respective mutant. However, this might only account for the Glu-CB1^−/−^ mutants, as all other animals, independently of line and genotype, displayed a similar memory and recognition performance. Especially after 24 hours, mice recognized and distinguished strongly between familiar and novel objects (Table 1). The low discrimination index to the familiar object after a 2 hour interval in several groups, however, is unexpected and cannot be explained at this point. Only Glu-CB1^−/−^ failed to show a clear preference towards the novel object in both retention sessions, indicating a memory deficit. Problematic for the interpretation is the overall low exploration for this mouse line, which is true for all three sessions of the novel object recognition test, as well as the other behavioural paradigms. Of special interest is the altered behaviour of the mutants in response to the novel objects. While wild-type littermates displayed a constant interest for the novel objects (O1–O3), the Glu-CB1^−/−^ animals showed a steadily decreasing exploration over the three sessions (Fig. 1A,D,G). For both genotypes, such a decrease was seen regarding the exploration of the familiar objects (O1), which is not surprising, as novelty of this object strongly decreased with each session. Thus, the Glu-CB1^−/−^ mice appeared to respond to the familiar and novel object in a similar way, suggesting rather a habituation to the context than a memory deficit. Nevertheless, a final conclusion cannot be made.

As mentioned above, all groups, independently of the line and the genotype, showed a stronger preference for the social interaction partner as compared to the object in the sociability test (Table 1). This behaviour was expected, as animals normally prefer social over non-social contacts [25]. Surprisingly, we could not detect a significant preference towards the novel interaction partner in the social novelty phase (Table 1). While this preference was observed in several lines [25], in our hands it was only recognizable in the GABA-CB1^−/−^ mice. This finding could indicate that social discrimination is impaired in these mutants. However, comparable results from other studies suggest that a strong social preference does not necessarily predict a strong preference for social novelty. As a matter of fact, two different components of social behaviour were postulated to underlie sociability and social novelty, respectively. In addition, life history and development are responsible for lower or higher novelty preference [30].

Taken together the strong differences observed in the GABA-CB1^−/−^ and Glu-CB1^−/−^ animals in respect to their wild-type littermates might be explained by anxiolytic and anxiogenic responses to novelty, respectively. Nevertheless, the eCB system has also been shown to be involved in learning and memory function, which should be kept in mind here [31], [32]. It may be even likely that both anxiety and memory components function together in our paradigms, but to solve this issue would require further investigations using other behavioural paradigms.

Our results, namely the increase of exploration following the deletion of GABAergic CB1 receptor and the decrease of exploratory behaviour following the deletion of glutamatergic CB1 receptors, may explain the contradictory findings using Δ^9^-THC, URB597 and VDM11, as described in above. We suggest that increased or decreased exploratory drive, respectively, as response to cannabinoid treatment depends on the predominant modulation of either GABAergic or glutamatergic CB1 receptor, e.g. the activation of GABAergic CB1 receptor decreases exploration, while the activation of glutamatergic CB1 receptor leads to an increased investigatory drive. Thus, the decreased exploration induced by chronic and systemic activation of the eCB system with Δ^9^-THC might be due to the exogenous activation of the CB1 receptor in GABAergic interneurons [15]–[17]. The increased exploratory profile after inhibition of anandamide degradation or reuptake could be explained by a specific on-demand activation of the CB1 receptor on glutamatergic neurons [17]. On the other hand, the increased animate and inanimate interaction as a result of the complete deletion of the CB1 receptor might be caused by the increased GABAergic drive [7], [20]. It seems that the GABAergic drive is the predominant factor for behavioural outcome, when the eCB system is activated or blocked in a chronic manner. This makes the increased social interaction after URB597 treatment even more interesting, as in this case, the glutamatergic drive seems to be the predominant component. To test this hypothesis, Glu-CB1^−/−^ or GABA-CB1^−/−^ have to be injected with the respective drugs in comparable doses and tested in behavioural paradigms. Similar contradictory results were observed in pharmacological studies on anxiety and stress levels after cannabinoid administration, both being strongly involved in investigatory and exploratory drive [33], [34]. The opposite effects might also be based on cortical GABAergic or glutamatergic transmission. Therefore, depending on its specific spatiotemporal activation within neuronal circuits, this system can act as a major “bi-directional” neuromodulator [14], [34].

Our results might also be interesting in respect to some disorders, which are associated with inappropriate exploratory drive. Thus, a direct and indirect relation between these disorders and a dysregulation of GABAergic and/or glutamatergic transmission can be proposed. In animal models for autism, modulation of GABAergic transmission seems to be important [35], [36]. The induction of schizophrenia-like symptoms by administration of the NMDA receptor antagonist phencyclidine revealed an alteration of glutamatergic and GABAergic signalling in the prefrontal cortex [37]. Interestingly, the effects of phencyclidine could be blocked by CB1 receptor antagonist treatment [38]. It was further shown that down-regulation of cortical glutamatergic drive resulted in an increase in dopamine levels and a hyperactive phenotype, which could be blocked by cortical GABA receptor activation [39]. These findings indicate a cortical control in these neuronal disorders, caused also by imbalanced GABAergic and glutamatergic transmission, a mechanism also suggested by our findings. Recent publications even suggest glutamatergic, instead of dopaminergic transmission to be the major factor of schizophrenia [40].

In conclusion, our results indicate a major, but opposite role of the eCB system in cortical GABAergic and glutamatergic neurons in the regulation of exploration (Table 2). Hence, further investigations along this line should be able to detail the diverse effects of cannabinergic drugs on investigatory behaviour. As investigatory drive is often associated with impulsive behaviour, studies using respective paradigms would be of great interest. Lastly, in future studies, the regulatory properties of the eCB system on cortical excitatory and inhibitory drive should be exploited in psychiatric disorders, opening up a therapeutic avenue to restore a possible cortical imbalance pharmacologically.

**Table 2:** Summary of behavioural changes induced by conditional CB1 receptor deletion.

	Locomotion	Object Exploration	Social Exploration	Aggression
Wild-type	Normal	Normal	Normal	Normal
Glu-CB1^−/−^	Decreased	Decreased	Decreased	Increased
GABA-CB1^−/−^	Increased	Increased	Increased	Normal
D1-CB1^−/−^	Normal	Normal	Normal	Normal

“Normal” refers to similar to the wild-type behaviour on spontaneous locomotor activity (locomotion), investigation of object (object exploration) or of interaction partner (social exploration) and fights initiated (aggression).
